# The efficacy of a brief intervention in reducing hazardous drinking in working age men in Russia: the HIM (Health for Izhevsk men) individually randomised parallel group exploratory trial

**DOI:** 10.1186/1745-6215-12-238

**Published:** 2011-11-04

**Authors:** Elizabeth Allen, Olga Polikina, Lyudmila Saburova, Jim McCambridge, Diana Elbourne, Sergey Pakriev, Nadezhda Nekrasova, Maxim Vasilyev, Keith Tomlin, Alexey Oralov, Artyom Gil, Martin McKee, Nikolay Kiryanov, David A Leon

**Affiliations:** 1 Medical Statistics Department, London School of Hygiene & Tropical Medicine, (Keppel Street), London, (WC1E 7HT), UK; 2 Department of Non-Communicable Disease Epidemiology, London School of Hygiene & Tropical Medicine, (Keppel Street), London, (WC1E 7HT), UK; 3 Department of Social and Psychological Researches, Izhevsk State Technical University, (Studencheskaya Street), Izhevsk (426069), Russia; 4 Department of Social and Environmental Health Research, London School of Hygiene & Tropical Medicine, (Keppel Street), London, (WC1E 7HT), UK; 5 Department of Psychiatry, Izhevsk State Medical Academy (Let Pobedy Street.), Izhevsk (426054), Russian Federation; 6 Municipal School, (Lenin Street), Izhevsk (426 000), Russia; 7 Department of polyclinic therapy, Izhevsk State Medical Academy, (Kommunarov Street), Izhevsk, (426034), Russia; 8 I.M. Sechenov Moscow Medical Academy, (Trubetskaya Street), Moscow, (119991), Russia; 9 Department of Health Services Research and Policy, London School of Hygiene & Tropical Medicine, (Keppel Street), London, (WC1E 7HT), UK; 10 Vice Rector, Izhevsk State Medical Academy, (Kommunarov Street), Izhevsk, (426034), Russia

## Abstract

**Background:**

Russia has particularly low life expectancy for an industrialised country, with mortality at working ages having fluctuated dramatically over the past few decades, particularly among men. Alcohol has been identified as the most likely cause of these temporal variations. One approach to reducing the alcohol problem in Russia is 'brief interventions' which seek to change views of the personal acceptability of excessive drinking and to encourage self-directed behaviour change. Very few studies to evaluate the efficacy of brief interventions in Russia have been conducted. Motivational Interviewing (MI) is a person-centred counselling style which can be adapted to brief interventions in which help is offered in thinking through behaviour in the context of values and goals, to decide whether change is needed, and if so, how it may best be achieved.

**Methods:**

This paper reports on an individually randomised two-armed parallel group exploratory trial. The primary hypothesis is that a brief adaptation of MI will be effective in reducing self-reported hazardous and harmful drinking at 3 months. Participants were drawn from the Izhevsk Family Study II, with eligibility determined based on proxy reports of hazardous and harmful drinking in the past year. All participants underwent a health check, with MI subsequently delivered to those in the intervention arm. Signed consent was obtained from those in the intervention arm only at this point. Both groups were then invited for 3 and 12 month follow ups. The control group did not receive any additional intervention.

**Results:**

441 men were randomised. Of these 61 did not have a health check leaving 190 in each trial arm. Follow up at 3 months was high (97% of those having a health check), and very similar in the two trial arms (183 in the intervention and 187 in the control).

No significant differences were detected between the randomised groups in either the primary or the secondary outcomes at three months in the intention to treat analyses. The unadjusted odds ratio (95% CI) for the effect of MI on hazardous and harmful drinking was 0.77 (0.51, 1.16). An adjusted odds ratio of 0.52 (0.28, 0.94) was obtained in the pre-specified per protocol analysis.

**Conclusions:**

This trial demonstrates that it is possible to engage Russian men who drink hazardously in a brief intervention aimed at reducing alcohol related harm. However the results with respect to the efficacy are equivocal and further, larger-scale trials are warranted.

**Trial Registration:**

ISRCTN: ISRCTN82405938

## Background

Russia has one of the lowest life expectancies among industrialised countries [1]. In 2008, for males it was only 62 years [2]. Over the past 25 years life expectancy has fluctuated dramatically, driven largely by deaths among working age men whose pattern implicates alcohol as a major factor [3,4]. A case-control study [5] specifically identified the role of hazardous drinking patterns, including extended periods of binge drinking known as *zapoi *(episodes of two or more days of continuous drunkenness), and consumption of non-beverage alcohols (manufactured alcohol-containing substances not intended to be drunk [6]). In 2006 the Russian government imposed restrictions on the manufacture and sale of ethanol [7]. However, the scale of the problem means that there is still much to be done [8,9].

Beyond the need for policies aimed at reducing availability and affordability of alcohol, there is also an urgent need to develop more effective individual-level treatments. In Russia the treatment of alcohol problems is highly medicalised [10], and mainly delivered through specialist institutions (narcology dispensaries). While the available treatments include ones described as psychotherapeutic, they are highly directive. They include a procedure known as "coding" whereby the patient is persuaded by a doctor that he or she has been administered an agent which will cause them to become very ill if they drink alcohol [11]. While coding is regarded as a means of inducing a placebo effect, active pharmacological interventions such as disulfiram that produce unpleasant reactions if the person drinks are also employed [11]. The aim of most treatments in Russia is to achieve a "cure" or complete abstention, rather than harm reduction [12]. There has been little use of person-centred individual counselling. Treatment at narcology dispensaries or psychiatric hospitals is usually without charge to the patient, many being admitted either because of an acute medical or psychiatric episode induced by alcohol or they are required to undergo treatment by the courts as a result of having been charged with an offence. There is also a relatively developed private sector for treatment of alcohol problems, although this will only be affordable to a minority of the population. It is unknown how far effective interventions could be delivered outside the specialist treatment services.

'Brief' interventions for reducing levels of alcohol consumption and alcohol-related harm have been developed and implemented in many countries [13]. These seek to change views of personal acceptability of excessive drinking and to encourage self-directed behaviour change. They include simple forms of structured advice and brief counselling. Easy access to these interventions is possible as they can be delivered by a wide range of generic practitioners.

An international evidence-base has accumulated over more than 20 years, with efficacy data originating mainly from English speaking countries. Reductions in volume of alcohol consumed are typically about 10-15%[13] and reductions in the proportions of hazardous drinkers are between 10-19%[14]. Reductions in alcohol problems of a similar magnitude and in health service utilisation have also been identified [15]. Motivational Interviewing (MI), defined as "a facilitative, patient-centred counselling style for helping people explore and resolve ambivalence"[16], has become increasingly prominent within this literature.

Very little is known about the salience and applicability of these interventions in Russia. In the1980s an international project undertaken by the World Health Organization involved a randomized trial of brief interventions to reduce alcohol-related problems [17]. This reported evidence of efficacy in reducing hazardous drinking among men in the Russian (Moscow) centre who were recruited either through hospital clinics or workplace health checks. This study was conducted at the height of the Gorbachev anti-alcohol campaign in the Soviet Union [18], which, together with other aspects of the study, means that interpretation of the trial results is problematic. The only other report of a trial of a brief intervention in Russia we have found is a protocol for a multi-arm randomised trial, including brief intervention aimed at reducing alcohol use and harms among TB patients in Tomsk, Siberia [19]. This trial is ongoing and results have yet to be published.

The aim of the Health of Izhevsk Men (HIM) study was to explore the efficacy and acceptability of a brief intervention aimed at reducing the prevalence of hazardous and harmful drinking in working age men in Izhevsk. This is on the Western-side of the Urals with a demographic profile typical of medium-sized Russian cities. The HIM study aims to prepare the ground for subsequent effectiveness evaluations in a range of routine service settings.

## Materials and methods

The study was an individually randomised two-armed parallel group exploratory trial. The Methods have already been described in detail [20], but are summarized below.

### Hypothesis

A brief adaptation of MI (referred to as MI) would be effective in reducing self-reported hazardous and harmful drinking in the previous month by 3 months post entry in the trial.

### Source population and data collection

The men recruited into the trial were drawn from a longitudinal observational study (the Izhevsk Family Study II). This was based on 1750 men who were the controls in a case-control study of premature mortality conducted 2003-5 [5] supplemented by a further 250 men recruited using an identical protocol in 2006. To avoid confusion, these controls, and the supplementary group of 250 men, are referred to jointly as index men.

At initial recruitment to the case-control study, the index men were aged 25-54 years and resident in Izhevsk and had been drawn at random from a population register. Interviews were conducted with proxy informants living in the same household as well as with index men themselves. Men living alone were not included. Interviewer-administered, structured questionnaires were used to gather information on a wide range of behaviours and characteristics including alcohol consumption. In 2008-9 we attempted to re-contact all of the index men who were still living in Izhevsk. Those who were successfully followed-up were asked if they, and a proxy informant living in the same household (if available), were prepared to be re-interviewed. As in the original case-control study (2003-5), proxy informants were mainly wives, but also included mothers, fathers and children of the men.

At the end of the re-interview, the index men were invited to have a "health check". This was scheduled to be carried out a few weeks later either at a polyclinic or in a minority of instances, their own home, according to the participant's preference.

The health check involved the doctor taking a medical history, measuring blood pressure, height and weight and taking a blood sample which was used to determine levels of the liver enzyme gamma-glutamyltransferase (GGT), a proxy marker for heavy alcohol drinking [21]. The man was also given a self-completed questionnaire that included the 10-item WHO Alcohol Use Disorders Identification Test (AUDIT)[22], modified to have a reference period of 3 months (instead of 1 year), to provide a meaningful outcome at the 3 month follow-up.

All of the information about alcohol problems described above was from self or proxy report. We also collected information at the time of the initial study (2003-5), from the local narcology dispensary, about whether each man had been treated with an alcohol-related primary diagnosis. This provides an objective and highly specific, although not very sensitive, marker of having had an alcohol problem.

### Trial inclusion criteria

Eligibility was determined by information about hazardous and harmful drinking gathered at the initial re-interviews with proxy informants. These criteria were: *zapoi *in the last year; drinking surrogates (non-beverage alcohols) in the last year; hangover and/or excessive drunkenness and/or going to sleep clothed due to being drunk twice or more per week on average over the past year; weekly consumption of 250 ml or more of ethanol (from beverages) over the past year. Men who lived alone at re-interview, or for whom no proxy interview could be obtained, were recruited on the basis of self reports of the same measures and using the same cut-offs.

### Trial exclusion criteria

Refusal to have a baseline health check and/or refusal at baseline re-interview to be followed up resulted in exclusion from the trial.

### Randomisation and consent

Data collected at the baseline re-interview were sent to the randomisation service in London, allowing participants to be allocated in a 1:1 ratio to MI intervention or no MI. Minimisation criteria (age, surrogate use in past year, and living alone status) were used to ensure a reasonable balance of confounding factors. An online randomisation program was used to generate the random allocation. Consent was obtained differently for the two groups (single consent Zelen design [23]).

#### (a) MI Intervention group

At the end of the health check, the doctor undertaking the physical examination opened a sealed envelope containing the allocation. For men allocated to MI, the doctor asked whetherhe would be prepared to attend a series of sessions at which his drinking would be discussed in a helpful way. Those who were willing were given an information sheet about the trial, and were given an opportunity to ask questions. If they agreed to take part, signed consent was sought for (i) participating in the intervention and (ii) providing follow up data in 3 and 12 months time;

#### (b) Control group

Telling the control group about the alcohol-specific MI intervention and the alcohol-specific outcomes, would have sensitised the control group to our primary research interest and thereby in itself may have altered behaviour. This could have also diluted any effect of the MI intervention [24]. Therefore consent to take part in the trial was not sought from men randomised into the control group. However all men had previously given general consent to be followed up at the time of the initial re-interview.

### MI practitioners

The acceptability of a brief intervention such as MI to professionals in Russia dealing with alcohol problems cannot be assumed. This is because the approach implicit in this type of brief intervention is in key respects contrary to the dominant model of alcohol treatment in Russia described in the Introduction: clients are seen as responsible for, and capable of, generating solutions to their own problems in MI, regardless of whether this is achieved through complete abstention or not.

We therefore chose to work outside of the conventional institutional setting to try and ensure that the content of the intervention was not distorted by the prevailing alcohol treatment paradigm.

We sought to identify potential practitioners who could work in the trial by holding training courses on MI. These were open to people from a wide range of professional backgrounds including narcologists (specialists in treating alcohol and drug dependency), psychiatrists, social workers and school psychologists. These courses elicited considerable interest, and 45 people participated in the initial 3-day course. From this group we identified 4 practitioners who were given further training and supervision in Russian, with a period of practice-based learning following an introductory workshop. A sample of sessions was audio-recorded for quality control and supervision purposes. In the end almost all of the sessions were delivered by one of two practitioners (a general psychiatrist, and a psychologist).

### Nature of interventions

#### (a) Intervention group

An adaptation of MI was developed for the Russian context. This was based on a previous topic-based approach to structuring the discussion in each session (see Additional file 1) [25]. Eligible men who had consented at the end of the health check to participate in the intervention were contacted to arrange their first session by an MI practitioner. The intervention comprised up to four sessions, the first two of which were protocol driven, with an additional two sessions available on request. These were delivered at a clinic or at home with the two core sessions being approximately two weeks apart.

#### (b) Control group

This group did not receive any intervention other than having a health check and the general health promotion feedback in the form of a letter that the intervention group also received.

### Outcome measures

Men in both intervention and control groups were contacted again to take part in a 3 and 12 month follow-up, measured from the time they had their initial health check examination. Outcome interviews were partly interviewer-administered and partly self-completed by participants.

The primary outcome was self-report of hazardous and harmful drinking at three months defined as: one or more occurrences of *zapoi *in the past month; surrogates in the past month; hangover on average twice or more per week over the past month; going to sleep clothed due to being drunk on average twice or more per week over the past month; or 250 mls or more of ethanol from beverages in the past week from beverages (i.e. 25+ UK alcohol units). The primary outcome was measured at 3 months as the effects of brief interventions are known to decline over time [26] and in the context of this study it was judged important to establish whether there was any evidence of effectiveness in Russia. The secondary outcomes considered in this paper are the separate components of the primary outcome. The 12 month outcomes will be reported separately.

### Payment to participants

Participants were paid a small sum of money in cash (100 roubles ≈ £2) to cover transport costs and for their time whenever they were interviewed (at baseline and at 3 month follow-up), when they attended the health check examination and at each MI session they attended.

### Sample size

Based on the brief interventions literature, it was expected that 25% of participants in the intervention arm would stop hazardous and harmful drinking. We allowed for regression to the mean of approximately 5% in the control group in this Zelen design [26]. Power calculations were therefore based on detecting a 20% difference between randomised groups (95% vs. 75%) with 90% power at the 5% level of statistical significance. This yielded a target sample size of 130 men (65 in each arm). We assumed that 20% of those allocated MI would not agree to receive the intervention. We also assumed a 20% loss to follow for the 3 month assessment in both trial arms. This required inflating the sample size to approximately 200 participants.

### Type of analysis

Participants were identified by their trial number to ensure confidentiality. The primary analysis was based on a difference in the number of men classified as hazardous and harmful drinkers at the 3 month assessment between the randomised groups using the intention to treat principle. Analyses were also adjusted for key prognostic factors. An error in the algorithm for ethanol consumption that led to over-estimation of weekly consumption meant that 11 men in the MI group and 16 in the no MI group were randomised, although ineligible. They were included in table 1 and in the intention to treat analyses. In addition, the baseline AUDIT score was included in an exploratory analysis due to an apparent imbalance between randomised groups at baseline. Indicative differential effects by subgroup analyses based on important prognostic factors such as age group and severity of alcohol dependence were assessed using interaction tests. A per-protocol analysis was defined based on those in the intervention arm who received at least 2 sessions of MI before the 3 month interview was undertaken.

**Table 1:** Baseline characteristics at randomisation

Baseline characteristics* at randomisation	Random allocation			
	
	MI N = 221		No MI N = 220	
	
	N	(%)	N	(%)
Age and living situation				

Age (years)				
30-40	47	(21.3)	48	(21.8)
> 40 < = 50	68	(30.8)	66	(30.0)
51-59	106	(48.0)	106	(48.2)

Lives alone	4	(1.8)	4	(1.8)

Proxy report (self report* if lives alone) of alcohol drinking				

Hazardous drinking in the last year	210	(95.0)	204	(92.7)

Surrogates in the last year	47	(21.3)	42	(19.1)

*Zapoi *in the last year	70	(31.7)	64	(29.2)

Hangover/excessive alcohol/bed clothed twice or more per week on average over past year	33	(14.9)	28	(12.7)

Average weekly consumption of ethanol over past year > 250 ml	170	(76.9)	162	(73.6)

Self report of alcohol drinking				

Hazardous drinking in the last year	157	(71.0)	159	(72.3)

Surrogates in the last year	32	(14.5)	29	(13.2)

*Zapoi *in the last year	35	(16.0)	41	(18.7)

Hangover/excessive alcohol/bed clothed twice or more per week on average over past year	8	(3.6)	9	(4.1)

Average weekly consumption of ethanol over past year > 250 ml	124	(56.1)	127	(57.7)

AUDIT Score **				
Level 1	64	(35.8)	44	(24.3)
Level 2	67	(37.4)	89	(48.9)
Level 3	24	(13.4)	21	(11.5)
Level 4	24	(13.4)	28	(15.5)
Missing	41		39	

* Based on proxy report, if available; otherwise self report. Of the allocated to the MI (intervention arm) 17/221 were allocated based on self-report. The corresponding figures for the non-MI (control arm) were 15/220.

** Intervention recommended for AUDIT scores

Level 1; Alcohol Education

Level 2: Simple Advice

Level 3: Simple Advice plus Brief Counselling and Continued Monitoring

Level 4: Referral to Specialist for Diagnostic Evaluation and Treatment

It had been planned that an independent Data Monitoring Committee would review data for the 3 month outcomes from the trial approximately 12 months from the start of the recruitment period. Recruitment was so rapid, however, that this interim analysis had to be abandoned. Recruitment to the trial continued until all men who were successfully followed-up had been assessed for eligibility and randomised into the trial if appropriate.

## Results

**Figure 1:**
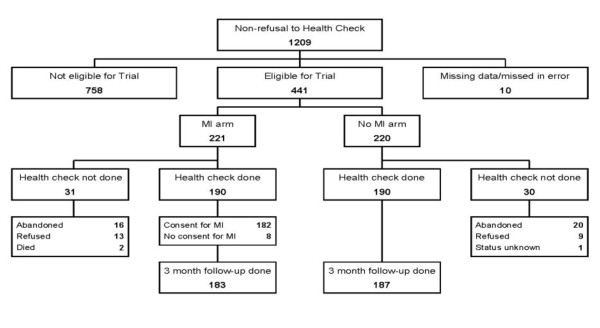
**Flow chart showing source and allocation of trial participants**.

A total of 1515 men were initially interviewed in 2008-9 as part of the Izhevsk Family Study II. All of these men were offered a subsequent health check. Figure 1 shows the flow of the 1209 men through the trial process who at this initial stage did not refuse to have a health check. Of the 441 randomised, 61 did not have a health check (31 in the intervention arm and 30 in the control arm), leaving 190 in each trial arm. Follow up at 3 months was very high (97% of those having a health check), and similar in the two trial arms (183 in the intervention and 187 in the control).

Table 1 shows the baseline characteristics of all the participants randomised to the trial. It also includes the self reported measures at baseline for comparison with the self reported 3 month outcomes.

The two groups were very similar at baseline except for AUDIT score. Ten percent of participants in both arms were known to have been treated at the narcology dispensary. The median [IQR] GGT was 35.1 [22.6, 63.6] in the control arm and 31.8 [21.9, 60.2] in the intervention arm.

Nearly 70% (n = 131) of those allocated to MI who had a health check had at least one session, and nearly 60% (n = 113) had at least two sessions. However, due to logistical problems, fewer than 40% had both these sessions prior to the follow up 3 months after the health check (Table 2).

**Table 2:** Adherence with protocol among 190 men in the MI group who had initial health check

Adherence with protocol*	N	(%)
At least 1 MI session	131	(68.9)

At least 2 MI sessions	113	(59.5)

At least 2 MI sessions and both dates available	109	(57.4)

Both MI sessions before 3 months follow up	72	(37.9)

First MI sessions before 3 months follow up but second session after 3 months follow up	28	(14.7)

Both MI sessions after 3 months follow up	9	(4.7)

* Definition for per protocol analysis is 2 MI sessions carried out between date of health check and date of 3 month follow up

No significant differences were detected between the randomised groups in either the primary or the secondary outcomes at three months in the intention to treat analyses (Table 3). The unadjusted odds ratio (OR) for the effect of MI on hazardous and harmful drinking (95% confidence interval (CI)) was 0.77 (0.51, 1.16). A sensitivity analysis excluding the 27 men randomised based on the erroneous estimate of baseline ethanol consumption did not affect these results. Adjustments for the baseline values of the outcomes and the imbalance in AUDIT score reduced the OR (95% CI) to 0.64 (0.39, 1.06).

**Table 3:** Outcomes at 3 months follow up

	Random allocation				Intention to treat			Per protocol
	
	MI N = 183		No MI N = 187		Unadjusted OR (95% CI)	Adjusted * OR (95% CI)	Adjusted ** OR (95% CI)	Adjusted ** OR (95% CI)
					
	Baseline n (%)	3 months n (%)	Baseline n (%)	3 months n (%)				
Primary outcome								
Hazardous drinking over previous month	129 (70.5)	87 (47.5)	138 (73.8)	101 (54.0)	0.77 (0.51,1.16)	0.79 (0.52,1.19)	0.64 (0.39,1.06)	0.53 (0.29,0.97)

Components of primary outcome								

Surrogates over previous month	22 (12.0)	9 (4.9)	25 (13.4)	11 (5.9)	0.83 (0.33,2.04)	0.87 (0.31,2.46)	0.77 (0.23,2.54)	0.23 (0.02,2.32)

*Zapoi *over previous month	30 (16.4)	13 (7.1)	33 (17.7)	13 (6.7)	1.03 (0.46,2.29)	1.08 (0.47,2.49)	0.85 (0.31,2.34)	0.50 (0.12,2.01)

Hangover/bed clothed twice or more per week on average over previous month ***	7 (3.8)	14 (7.7)	9 (4.8)	11 (5.8)	1.33 (0.59,3.02)	1.39 (0.61,3.16)	0.95 (0.37,2.46)	1.20 (0.39,3.67)

Average weekly consumption of ethanol over past month > 250 ml	103 (56.3)	83 (45.4)	112 (59.9)	90 (48.1)	0.89 (0.59,1.35)	0.92 (0.60,1.39)	0.77 (0.47,1.26)	0.68 (0.37,1.25)

* adjusted for baseline values of outcome measures (or similar) using analysis of covariance

** additionally adjusted for AUDIT score

*** excessive drinking not asked at 3 month

Pre-specified sub-group analyses based on the different age groups, extent of hazardous and harmful drinking as measured by the AUDIT score, GGT, and narcology registration did not detect any significant difference in the effect of MI on hazardous and harmful drinking at three months (interaction tests p = 0.28 (age groups), 0.88 (AUDIT score, 0.78 (GGT) and 0.73 (narcology registration)).

The per-protocol analysis gave an odds ratio of 0.52 (0.28, 0.94), after adjustment for self reported hazardous and harmful drinking at baseline and the AUDIT score.

## Discussion

This exploratory trial aimed to assess the acceptability (to participants and professionals) of a brief intervention for reducing alcohol consumption and harms among men in Russia, as well as providing some indication of potential efficacy.

### Acceptability

This trial is the first to demonstrate that in Russia today it is possible to engage men across the full spectrum of drinking problems identified in the community in a brief intervention aimed at reducing alcohol related harm. Almost 70% of those offered MI attended a first session, with 60% going on to have two or more sessions. However, the extent to which this experience is fully generalisable is not clear as the men who took part in the trial had already been acquainted with the research team from their participation in the earlier observational studies that had begun in 2003.

In setting up the trial it became apparent that there was very limited initial understanding among the health and other professionals of the value of evidence generated from randomised trials. This has to be understood against a background of the rejection of randomisation by Soviet science [27]. Even today there is relatively little expertise in the conduct or analysis of RCTs apart from in the pharmaceutical industry in Russia, with the principles of evidence-based medicine and practice being largely absent from the medical curriculum in Russia [28].

### Fidelity of intervention

As the main trial proceeded, it became evident that it was difficult for some of the potential practitioners to continue to be engaged because the trial was not embedded within their work institution. Regular supervision of the two remaining practitioners by one of the authors (OP) involved discussions of sessions based on audio-recordings. These suggested considerable difficulties in applying the more sophisticated and advanced features of MI. It is thus likely that MI was not consistently delivered to international standards. In this context it is interesting to note that the per protocol analysis suggested a positive effect of the intervention if delivered in advance of the 3 month health check. The low level of intervention delivery within the three month follow-up study makes more important evaluation of outcome data for efficacy purposes at the later twelve month follow-up study.

### Efficacy

The results of this exploratory trial with respect to the efficacy of MI are equivocal. The study was powered to detect a relatively large effect of a 20% difference between intervention and control (75% vs 95%) in the prevalence of hazardous and harmful drinking at 3 month follow-up. However, in the main intention to treat analysis we observed a much smaller 6.5% difference (47.5% vs 54%) that was not statistically significant. The per-protocol analysis showed a slightly larger effect after adjustment for baseline differences between control and intervention. However, these latter analyses need to be interpreted with caution; although overall the data are consistent with the intervention being effective, the size of the true effect was probably smaller than we had anticipated.

There were a number of problems with the implementation of the protocol which could contribute to the lack of a clear effect being identified. Firstly, there were delays in scheduling the MI sessions, which were primarily due to the MI practitioners having to fit this in on top of their full time professional commitments. Just under 40% of the men had received 2 or more MI sessions prior to the 3 month outcome interview, while 4.7% had their first session after the 3 month check. This aspect of the trial design would have diluted any effect of the intervention (assuming the intervention was actually effective). Even without these trial-specific timing constraints a dilution of effect was likely given that only 60% of men eventually had two MI sessions, which is itself an important acceptability finding.

A further factor that needs to be taken into account is that the trial was conducted within a population that has been repeatedly contacted in our previous observational studies since 2003. As a result all the men in the trial may have become particularly sensitised to general health issues following the health check feedback given to all participants. Moreover, all men regardless of trial arm completed the AUDIT and answered other alcohol questions that may have led them (and their partners) to reflect on their drinking behaviour, thus potentially having an effect on the behaviour of the control as well as the intervention arms.

It could be expected that MI delivered with a greater level of fidelity than was possible here would yield greater evidence of efficacy. However, the experience of the delivery of interventions in this trial more closely resembles an effectiveness study in which the effect of any difficulties practitioners may have in learning and applying a new method becomes part of the object of evaluation. An alternative way to interpret the outcome data, therefore, is to consider them as representing the likely effects of a generic brief intervention directed at hazardous and harmful drinking, which has been variably implemented, rather than the specific effects of MI *per se *in an efficacy context.

Finally, it should be noted that we intentionally included all men whom we classified as hazardous and harmful drinkers based on proxy reports at baseline. To our knowledge there are no previous trials of brief interventions which have used this recruitment method. This included a proportion of men who undoubtedly had long histories of heavy drinking and were alcohol dependent. It is known however that effect sizes for brief interventions are smaller when, as in this study, dependent drinkers are not excluded [26].

### Implications for future research

We suggest that any future trials should be suitably powered to detect whether this type of intervention is similarly effective in men with established and profound alcohol problems compared to those who are drinking hazardously but have less serious problems. The decision not to situate the intervention within an institutional setting led to MI practitioners having to fit in MI sessions around their full-time work leading to delays in scheduling the MI sessions. Future trials will therefore need to be embedded within institutional frameworks to ensure that those working on the trial are able to integrate this into their routine work making it more likely that this type of intervention is subsequently incorporated into routine practice if shown to be effective.

## Conclusions

This trial demonstrates that it is possible to engage Russian men who drink hazardously in a brief intervention aimed at reducing alcohol related harm. However the results with respect to the efficacy are equivocal and further, larger-scale trials are warranted.

## Competing interests

The authors declare that they have no competing interests.

## Authors' contributions

Design: DE, DAL, JM, MM; Study fieldwork: AG, NK, OP, LS, MV; Motivational interviewing training: JM, OP; Intervention: NN, SP supervised by OP and JM. Data management: AO, KT; Randomisation and statistical analysis: EA who also wrote the first draft of the paper. All authors commented on and contributed to subsequent drafts of the paper. DAL is the study guarantor. All authors read and approved the final manuscript.

## Supplementary Material

Additional file 1 **Intervention Protocol**. This is the protocol used for the intervention. Click here for file

## References

[B1] McMichael AJ McKee M Shkolnikov V Valkonen T Mortality trends and setbacks: global convergence or divergence? Lancet 2004 363 9415 1155 9 10.1016/S0140-6736(04)15902-3 15064037

[B2] Leon DA Trends in European life expectancy: a salutary view Int J Epidemiol 2011 40 2 271 7 10.1093/ije/dyr061 21415000

[B3] Leon DA Chenet L Shkolnikov VM Zakharov S Shapiro J Rakhmanova G Huge variation in Russian mortality rates 1984-94: artefact, alcohol, or what? Lancet 1997 350 9075 383 8 10.1016/S0140-6736(97)03360-6 9259651

[B4] Leon DA Shkolnikov VM McKee M Alcohol and Russian mortality: a continuing crisis Addiction 2009 104 1630 6 10.1111/j.1360-0443.2009.02655.x 19681805

[B5] Leon DA Saburova L Tomkins S Andreev E Kiryanov N McKee M Hazardous alcohol drinking and premature mortality in Russia: a population based case-control study Lancet 2007 369 9578 2001 9 10.1016/S0140-6736(07)60941-6 17574092

[B6] Gil A Polikina O Koroleva N McKee M Tomkins S Leon DA Availability and characteristics of nonbeverage alcohols sold in 17 Russian cities in 2007 Alcohol Clin Exp Res 2009 33 1 79 85 10.1111/j.1530-0277.2008.00813.x 19018753

[B7] Levintova M Russian alcohol policy in the making Alcohol Alcohol 2007 42 5 500 5 17537829 10.1093/alcalc/agm040

[B8] Gil A Polikina O Koroleva N Leon DA McKee M Alcohol policy in a Russian region: a stakeholder analysis Eur J Public Health 2010 20 5 588 94 10.1093/eurpub/ckq030 20350932 PMC2943508

[B9] Khaltourina DA Korotayev AV Potential for alcohol policy to decrease the mortality crisis in Russia Eval Health Prof 2008 31 3 272 81 10.1177/0163278708320160 18662923

[B10] Fleming PM Drug and alcohol user treatment/intervention services in Russia--a Western perspective Subst Use Misuse 1996 31 1 103 14 10.3109/10826089609045801 8838396

[B11] Raikhel E Post-soviet placebos: epistemology and authority in Russian treatments for alcoholism Cult Med Psychiatry 2010 34 1 132 68 10.1007/s11013-009-9163-1 19967435

[B12] Elovich R Drucker E On drug treatment and social control: Russian narcology's great leap backwards Harm Reduct J 2008 5 23 10.1186/1477-7517-5-23 18577225 PMC2474597

[B13] Kaner EF Beyer F Dickinson HO Pienaar E Campbell F Schlesinger C Effectiveness of brief alcohol interventions in primary care populations Cochrane Database Syst Rev 2007 2 CD004148 10.1002/14651858.CD004148.pub3 17443541

[B14] Whitlock EP Polen MR Green CA Orleans T Klein J Behavioral counseling interventions in primary care to reduce risky/harmful alcohol use by adults: a summary of the evidence for the U.S. Preventive Services Task Force Ann Intern Med 2004 140 7 557 68 15068985 10.7326/0003-4819-140-7-200404060-00017

[B15] Fleming MF Mundt MP French MT Manwell LB Stauffacher EA Barry KL Brief physician advice for problem drinkers: long-term efficacy and benefit-cost analysis Alcohol Clin Exp Res 2002 26 1 36 43 10.1111/j.1530-0277.2002.tb02429.x 11821652

[B16] Miller WR Rollnick S Motivational Interviewing: Preparing People for Change 2002 New York: Guilford Press

[B17] Babor TF Grant M WHO Programme on Substance Abuse. Project on identification and management of alcohol-related problems Report on Phase II: A randomized clinical trial of brief interventions in primary health care 1992 Geneva: World Health Organisation

[B18] White S Russia Goes Dry 1996 Cambridge: Cambridge University Press

[B19] Greenfield SF Shields A Connery HS Livchits V Yanov SA Lastimoso CS Integrated Management of Physician-delivered Alcohol Care for Tuberculosis Patients: Design and Implementation Alcohol Clin Exp Res 2010 34 2 317 30 10.1111/j.1530-0277.2009.01094.x 19930235 PMC2898509

[B20] Tomkins S Allen E Savenko O McCambridge J Saburova L Kiryanov N The HIM (Health for Izhevsk Men) trial protocol BMC Health Serv Res 2008 8 1 69 10.1186/1472-6963-8-69 18377650 PMC2364619

[B21] Conigrave KM Davies P Haber P Whitfield JB Traditional markers of excessive alcohol use Addiction 2003 98 Suppl 2 31 43 14984240 10.1046/j.1359-6357.2003.00581.x

[B22] Saunders JB Aasland OG Babor TF De lF Jr Grant M Development of the Alcohol Use Disorders Identification Test (AUDIT): WHO Collaborative Project on Early Detection of Persons with Harmful Alcohol Consumption--II Addiction 1993 88 6 791 804 10.1111/j.1360-0443.1993.tb02093.x 8329970

[B23] Zelen M Randomized consent designs for clinical trials: an update Stat Med 1990 9 6 645 56 10.1002/sim.4780090611 2218168

[B24] McCambridge J Day M Randomized controlled trial of the effects of completing the Alcohol Use Disorders Identification Test questionnaire on self-reported hazardous drinking Addiction 2008 103 2 241 8 10.1111/j.1360-0443.2007.02080.x 18199302

[B25] McCambridge J Strang J Development of a structured generic drug intervention model for public health purposes: a brief application of motivational interviewing with young people Drug Alcohol Rev 2003 22 4 391 9 10.1080/09595230310001613903 14660128

[B26] Moyer A Finney JW Swearingen CE Vergun P Brief interventions for alcohol problems: a meta-analytic review of controlled investigations in treatment-seeking and non-treatment-seeking populations Addiction 2002 97 3 279 92 10.1046/j.1360-0443.2002.00018.x 11964101

[B27] McKee M Cochrane on Communism: the influence of ideology on the search for evidence Int J Epidemiol 2007 36 2 269 73 10.1093/ije/dym002 17360779

[B28] Danishevski K McKee M Balabanova D Prescribing in maternity care in Russia: the legacy of Soviet medicine Health Policy 2008 85 2 242 51 10.1016/j.healthpol.2007.08.001 17854946

