# Supplementary material for: The efficacy of a brief intervention in reducing hazardous drinking in working age men in Russia: the HIM (Health for Izhevsk men) individually randomised parallel group exploratory trial
Source: Trials. 2011 Nov 4;12:238. doi: 10.1186/1745-6215-12-238 (PMC3222605; doi:10.1186/1745-6215-12-238)
Supplement: Additional file 1 — Intervention Protocol. This is the protocol used for the intervention. [file 1745-6215-12-238-S1.DOC]

INTERVENTION PROTOCOL

Session 1

1. Orientation

Explain allocation, study procedures, offering opportunities to ask questions. Provide standardised brief account of intervention and remind about the previously signed consent (at health check). If doesn’t remember or didn’t sign, provide with the Informed Consent form and ask to read and sign if agrees to proceed. Acquire verbal consent for the session to be tape-recorded.

1. Rapport-building

Initiate conversation about the broader life context on subjects which are neither complex nor sensitive. Communicate interest in person before any discussion of drinking by discussing work and (non-alcohol) leisure interests, home and family relationships etc. You do not have to cover all the above-mentioned subjects, if you’ve identified areas the participant finds easy to talk about and he’s started talking to you willingly. The main purpose is to quickly create an atmosphere in which the participant will feel comfortable discussing drinking and its’ consequences. The secondary purpose is to gather background information for later use. End by asking a general open question about drinking. If the answer differs from information you were given by research team, at this moment do not try to “catch out” the participant.

1. Good and less good things about drinking

Invite the participant to tell you what is good about their drinking before any discussion of the ‘less good’ things. Encourage elaboration of positives even if he starts saying something like “I know there’s nothing good in it…or it’s no big deal” etc. Invite similar consideration of negative aspects of drinking, being careful about terminology used. The objective is to encourage the participant to articulate as far as possible how they really see and evaluate their own behaviour. This discussion may also alert both participant and practitioner to areas of doubt and uncertainty as well as to obvious ‘problem’ issues. Simple reflections and open questions should be useful here and there should be plenty of material generated for summaries. Pay attention to giving them time and space, by taking care not to rush.

1. Values and goals

Invite the participant to tell you about what is important to them in their lives, not only goals and aspirations for the future but also valued activities, relationships etc that are current. This material will build upon information gathered in rapport-building. The purpose is to help the participant to make a strong statement of non-alcohol values and goals that may be compromised or jeopardised by hazardous drinking. Helping the participant to summarise is a good technique here.

1. Risks, problems and concerns

This exercise encourages the participant to consider whether there may be any potential discrepancy between their current behaviour and their values and goals. Introduce the concepts of risks, problems and concerns and invite participants to consider whether they see anything risky, problematic or concerning about their own drinking. The practitioner must specifically avoid interference with this activity which pushes the participant towards particular outcomes. They may raise material that has been earlier identified in the form of a question, but it is for the participant to decide how it should be categorized. Summaries and more complex reflections are particularly relevant here. Where problems and concerns are identified they should be explored to focus thinking upon precisely what is at issue.

1. Readiness, importance and confidence tools

Introduce ‘rulers’ as simple tools. Guide participant to helpful questions to be asking (why this score and not lower…what needs to happen for this score to move up). Invite participant to consider importance, then confidence and finally readiness. Do these scores accurately represent how the participant sees their situation? What are the implications of these scores?

1. Decisional balance exercise

Invite consideration of one possible change as an example of how one might think through a decision to change. The more active is the participant the better. Use pen and paper to construct a simple grid, starting with the status quo. Explicitly encourage the participant to introduce all material that has already been discussed, as well as new material. Use reflective listening throughout. The costs of change material may be used to move the discussion on to how change may actually be secured.

1. Ending (including arrangement of Session 2, homework)

The ending of the first session is particularly important. The aim is to optimally prepare the participant to make use of the time before the second session. This may take many forms and may or may not include the agreement of specific homework tasks. These should be genuinely individualised and targeted at particular issues that will benefit from ongoing attention. The practical arrangements for the second session should be made, perhaps following an indication of session content.

All 8 items should usually be covered during Session 1 within 1 hour. If it has been fruitful to explore particular issues in some depth and time is short, it will be better to omit items 6 and 7 from discussion than to rush the Ending (7 may also be straightforwardly converted into a homework task if short of time).

Session 2 Protocol

1. Re-engagement

This may be best done by generally enquiring about how things have been since Session 1. This can serve to re-establish rapport and act as a precursor to a focus on more specific follow-up of Session 1 content, including discussion of any agreed homework tasks. It is also possible to invite the participant to reflect back on whether the previous discussion was or wasn’t helpful and their comments may provide useful feedback with implications for the content of this session. Summaries of previous material will be an efficient means of re-introducing issues for discussion.

1. Health-check data feedback

The health-check data summary should be provided taking care not to heighten anxiety or resistance, and encouraging the participant to articulate their own reactions as much as possible. The more detailed these verbal responses the better.

Stress personal responsibility and freedom to choose what to make of this information. Acknowledge the limitations of the data available. For medically trained practitioners, it is of great importance not to give any technical appraisal of the lab results (liver enzymes) – simple language should be used as far as possible.

1. Questionnaire assessment feedback

The issues of risk and problem severity should be briefly re-introduced and the participant should be invited to say how they found completing the questionnaires. They should then be presented with the results of AUDIT and LDQ scores along with guidance given on their interpretation. The participant should be given an opportunity to reflect upon the meaning of each score and to say what they think about it before proceeding to the next one. The practitioner must avoid over-emphasising or exaggerating problems as this will produce resistance. After all questionnaire scores have been fed back, the participant should be offered a brief summary and a further opportunity to consider the overall picture.

1. Decision-making

Summaries of the content of both sessions to this point should be constructed. The more this is done by the participant the better, with the practitioner if possible in a supportive role adding any significant information that has been missed out. This presents the participant with an opportunity to evaluate their situation and consider what they might do about it. The practitioner should ask general questions which clearly ‘put the ball in the court’ of the participant. They should specifically avoid any sense in which they are pushing for a decision to change or any other particular outcome. If further issues are identified for discussion, these should be pursued.

1. Self-monitoring

Where the participant does not reach a decision to change their drinking, this must be accepted as a valid choice. The purposes of further discussion are to help avoid or minimise problems that may be caused to the participant or others around him. There are a number of different possibilities which all seek to promote increased awareness of harm, including diary methods in which both patterns of consumption and consequences are monitored, looking back to periods when drinking was very different, and focusing in-depth on specific heavy drinking episodes. The relevance of specific issues will have been established in earlier discussion.

1. Change-planning

Where the participant does reach a decision to change their drinking, this must be explored in detail. Is this the right change, what alternative possibilities are there? Exactly how is what is being proposed to be done? What potential difficulties are there? A brainstorming style is appropriate, so long as it is the participant who is doing the work! The practitioner should act as a coach, suggesting possible difficulties and encouraging the participant in thinking through how they may be resolved.

1. Ending

There should be no assumption made that additional sessions are necessary, nor that they are specifically encouraged. Optimism should be communicated, regardless of whether or not change has been planned. Sessions 3 and 4 are entirely optional, and may be useful for checking on progress in change or for more general review and monitoring purposes. The participant can decide in the session whether or not they would like to arrange a further session, or be made aware of how to arrange a further session by telephone. There is no pre-defined material for Sessions 3 & 4. Participants should be encouraged to take up further sessions only if they themselves decide that it will be helpful to them.
